# First report of *Trichogrammadanausicida* and *Trichogrammacacaeciae* reared from *Thaumatotibialeucotreta* eggs in Israel

**DOI:** 10.3897/zookeys.779.25674

**Published:** 2018-08-02

**Authors:** Roy Kaspi, Svetlana Kontsedalov, Murad Ghanim

**Affiliations:** 1 Department of Entomology, ARO, Volcani Center, Rishon LeZion 7505101, Israel Department of Entomology, ARO, Volcani Center Rishon LeZion Israel

**Keywords:** DNA barcoding, egg parasitoid, False codling moth, *Ricinuscommunis*, Trichogrammatidae

## Abstract

The egg parasitpoids *Trichogrammadanausicida* (Nagaraja) and *Trichogrammacacaeciae* (Marchal) (Hymenoptera: Trichogrammatidae), are reported for the first time in Israel. Moreover, our discovery of *T.danausicida* is the first report of this parasitoid species outside of India. The occurrence of those trichogrammatids was first discovered and documented in May 2016 during a survey of egg parasitoids of the False codling moth *Thaumatotibialeucotreta* (Lepidoptera: Tortricidae). The field survey was conducted on castor bean fruits (*Ricinuscommunis*) in the Israeli central coastal plain. The identity of the parasitoids was revealed by means of sequencing a portion of the cytochrome oxidase I gene (COI) of the studied parasitoids.

## Introduction

The False codling moth (*Thaumatotibialeucotreta* (Meyrick); i.e., FCM) (Lepidoptera: Tortricidae), native to African regions south of the Sahara, was first reported in Israel in 1984 on macadamia nuts (Wysoki 1986). It is a polyphagous pest that can develop on more than 70 host plants (CABI 2017). Furthermore, FCM is an important economic pest to many crop fruits in its native habitat, such as citrus, macadamia, avocado, peach, plum, corn, cotton, peppers, and more. The annual estimated loss to the Southern African citrus industry alone, caused by this pest, is approximately 8 million USD (Kirkman and Moore 2007). Among wild plants, the castor bean (*Ricinuscommunis*) serves as a preferred host plant for the FCM, providing fruits nearly all year round for FCM development and survival (Kirkman and Moore 2007, CABI 2017, CABI 2018). One of the most effective parasitoids for controlling FCM in South Africa is the egg parasitoid *Trichogrammatoideacryptophlebiae* (Nagaraja) (Newton 1988, Bedford et al. 1998, Moore and Hattingh 2012). Moreover, *T.cryptophlebiae*’s natural parasitism level can reach more than 80 percent of the FCM eggs. In such cases, the FCM population level is significantly reduced in citrus orchards (Moore and Hattingh 2012). *T.cryptophlebiae* was introduced to Israel in 1998 for controlling the FCM. More than 300,000 parasitoids were released in the Israeli central coastal plain; however, no recovery was reported to date “(Yael Argov, pers. comm.). Other reported egg parasitoids that attack the FCM are *Trichogrammatoideafulva* (Nagaraja) and *Trichogrammatoidealutea* (Girault) (CABI 2017). We were interested in investigating whether *T.cryptophlebiae* was established on FCM eggs in Israel, and if not, are other egg parasitoids attacking FCM eggs? Therefore, the objective of this study was to perform a field survey of FCM egg parasitoids in the Israeli central coastal plain.

## Materials and methods

**Figure 1–2.**
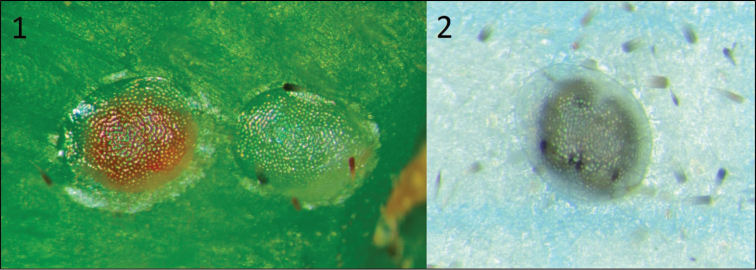
*Thaumatotibialeucotreta* eggs. **1** Unparasitised young (clear white) and mature (red) eggs **2** parasitised by *Trichogramma* spp.

A survey of FCM egg parasitoids was performed on castor bean plants (*Ricinuscommunis*) in the Israeli central coastal plain (Table 1). The survey sites were determined based on the locations where *T.cryptophlebiae* were originally released in 1998, and where castor bean plants were found. Only sites where FCM eggs were actually found are shown in Table 1. Castor bean fruits were randomly collected from each site and transferred to the laboratory. The fruits were then carefully examined under a stereoscopic microscope, and the number of FCM eggs and their status were recorded. The egg status included: hatched eggs (i.e., egg shells), dead eggs, live eggs, or parasitized eggs (Figs 1–2). Parasitised eggs and suspected as such, were individually confined within petri dishes (55 mm in diameter, 26 mm height), and observed daily for adult emergence. After emergence, the adults were placed in 75% ethanol until their identity was determined using DNA sequencing.

**Table 1.** The Universal Transverse Mercator (UTM) coordinates of nine castor bean collection sites, and the number of FCM eggs that were found in each location.

Site	Latitude	Longitude	Elevation (m)	Total number of eggs
1	32°06'55"N, 34°54'20"E		22	84
2	32°09'52"N, 34°52'49"E		68	15
3	32°09'10"N, 34°54'25"E		39	63
4	32°20'44"N, 34°53'44"E		32	33
5	32°20'58"N, 34°52'30"E		31	764
6	32°00'15"N, 34°49'00"E		34	33
7	32°08'50"N, 34°53'04"E		33	17
8	31°59'10"N, 34°48'06"E		36	87
9	32°08'07"N, 34°53'27"E		19	45

DNA was extracted from single parasitoids in 25 μL lysis buffer (Skaljac et al. 2013). This DNA was used for amplification of 800 bp from the mitochondrial Cytochrome Oxidase I (COI) gene using Polymerase Chain Reaction (PCR). PCR was performed in a total volume of 50 μL containing 25 μL of Ready Mix (HyLabs, Israel), 13.5 μL double distilled water, 0.75 μL of 20 pmole for each primer used, and 10 μL of DNA template (total of 200 ng). The primer sequences used for PCR are LCO_1490F 5’-GGTCAACAAATCATAAAGATATTGG-3’ and HCO_2198R 5’-TAAACTTCAGGGTGACCAAAAATCA-3’. PCR cycling conditions were 94 °C for 5 min, followed by 35 cycles of 94 °C for 30 sec, 45 °C for 45 sec, and 72 °C for 1 min, with a final extension at 72 °C for 10 min.

*Trichogrammatoideacryptophlebiae* parasitoids obtained from South Africa (from Vital Bugs®, Tzaneen, South Africa) were tested with the same pair of primers mentioned above, however, the obtained sequences did not match any sequences in GenBank (www.ncbi.nlm.nih.gov/Genbank), thus, an additional pair of primers that amplify a portion of the Internal Transcribed Spacer 2 sequences (ITS 2), located in the 5.8S and 28S region of the rDNA complex bordering the ITS 2 region, were used. Their sequences are: ITS2-F 5’-TGTGAACTGCAGGACACATG-3’ and ITS2-R 5’-GTCTTGCCTGCTCTGAG-3’. The PCR conditions were as follows: 94 °C for 3 min, followed by 33 cycles of 94 °C for 40 sec, 55 °C for 1 min and 72 °C for 1 min, with a final extension period at 72 °C for 5 min (Wahner et al. 2008). Each PCR reaction was examined by electrophoresis and bands were visualised with UV light. PCR products were excised from the gel and purified using the Nucleospin Gel and PCR Clean-Up Kit (Macherey-Nagel, Germany). Purified PCR products were sequenced in both the forward and reverse directions (HyLabs, Rehovot, Israel).

Sequence alignment and phylogenetic analysis: Sequence alignments for COI gene sequences were performed with MUSCLE 3.7 (Edgar 2004) and the results were adjusted manually where necessary, to maximise alignment. The alignment data for each gene were used in maximum likelihood tree construction, using Kimura-2 parameter model (K2P) genetic distances (Kimura 1980). Both trees were generated using MEGA v.5 (Tamura et al. 2011) and branch support was estimated with 1000 bootstrap replicates. The nucleotide sequences used in this study for generating the phylogenetic tree have been deposited in GenBank under the accession numbers MH102404 to MH102410.

## Results

*Thaumatotibialeucotreta* eggs were found from November 2015 to December 2016 on castor bean fruit in the Israeli central coastal plain. In total, on 2200 fruits, we detected 1141 eggs, of which 449 were alive (i.e., 39.3%). In May 2016, in location number 5 (Table 2), we detected seven parasitised eggs of which only six hatched. These eggs accounted for 3.7 percent of all live eggs that were found during May 2016 in this location.

**Table 2.** Collection dates, and number of FCM eggs that were found in a field survey, in nine different locations in the Israeli central coastal plain.

Site	Collection date	Number of fruits	Total number of eggs	Number of live eggs	Number of parasitized eggs	Percentage of parasitized eggs from live eggs
1	November 2015	100	28	5	0	0
1	June 2016	100	56	19	0	0
2	April 2016	50	15	7	0	0
3	April 2016	50	9	4	0	0
3	June 2016	100	54	19	0	0
4	May 2016	200	33	16	0	0
5	May 2016	500	518	184	7	3.7
5	June 2016	300	246	161	0	0
6	June 2016	150	29	1	0	0
6	December 2016	100	4	2	0	0
7	July 2016	50	17	6	0	0
8	August 2016	200	62	4	0	0
8	October 2016	100	25	3	0	0
9	November 2016	100	33	10	0	0
9	December 2016	100	12	8	0	0
Total		2200	1141	449	7	

**Figure 3.**
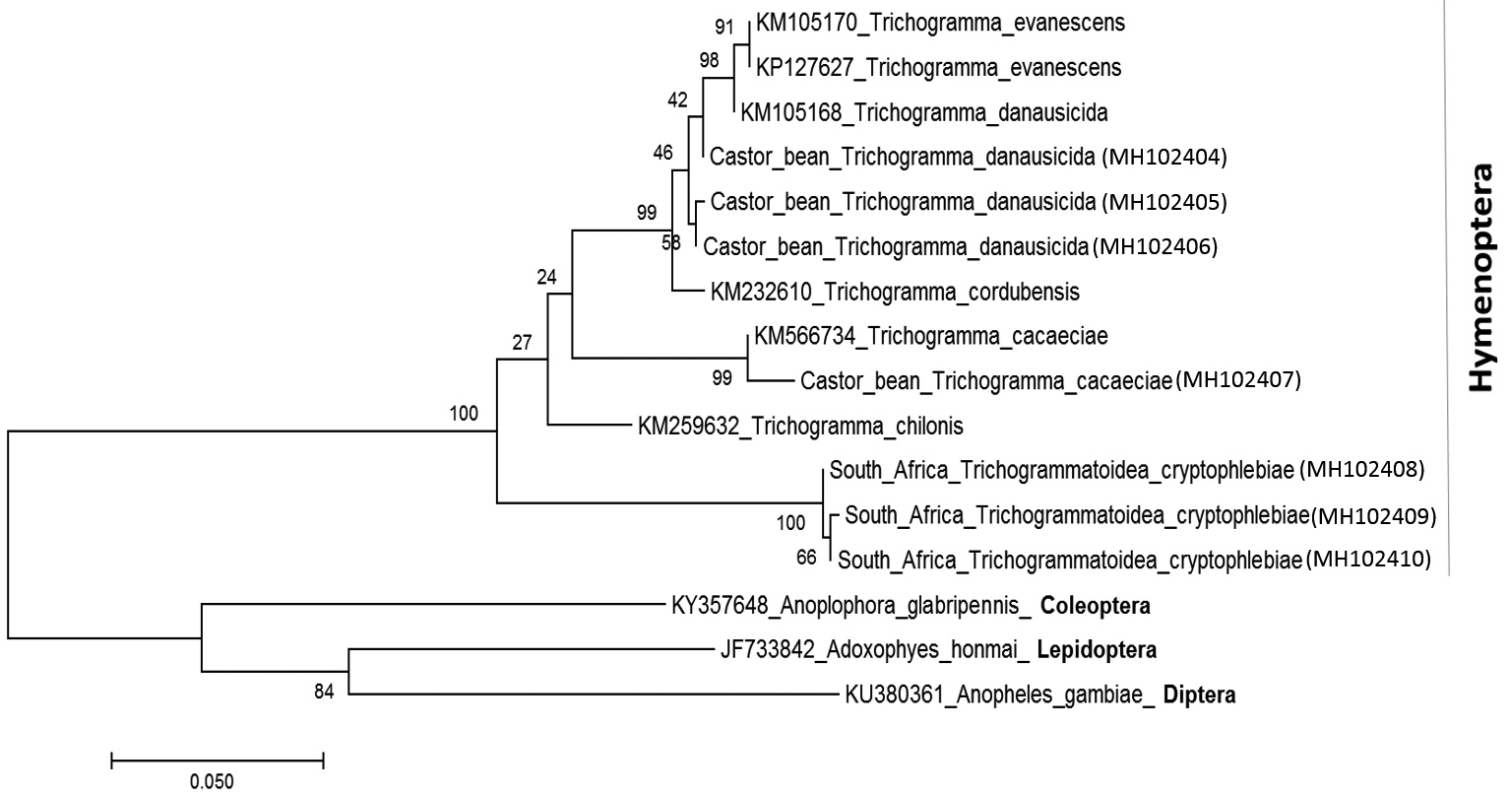
Maximum likelihood tree of COI nucleotide sequences of *Trichogrammadanausicida* and *Trichogrammacacaeciae* and other hymenoptera species. Other species from Coleoptera, Lepidoptera and Diptera were used as outgroups to construct the tree. The tree was constructed using Kimura-2 parameter model (K2P) genetic distances with MEGA v.5, and branch support was estimated with 1000 bootstrap replicates. Numbers in parentheses are accessions that were deposited in GenBank.

We sequenced a total of seven wasps (four specimens from Israel and three *T.cryptophlebiae* wasps from South Africa) and obtained their COI sequences. Those sequences were aligned with other Hymenoptera sequences and other outgroup sequences of species from other orders such as the Coleoptera, Diptera and Lepidoptera (obtained from GenBank). All species for which multiple specimens were sampled showed no interspecies variation. The maximum likelihood analysis of the COI gene resulted in a tree typology that showed the presence of two different species of trichogrammatids: *Trichogrammadanausicida* (Nagaraja)(3 specimens) (Nagaraja 1996) and *Trichogrammacacaeciae* (Marchal) (one specimen) (Marchal 1927) (Fig. 3), which were clearly separated, but fall within the Hymenoptera.

## Discussion

*Trichogramma* spp. are minute endoparasitoids of insect eggs. Currently, more than 230 species of *Trichogramma* are described worldwide, making them the largest genus in the Trichogrammatidae family. More than 200 insect species are being attacked by different *Trichogramma* species. Moreover, many species of Trichogramma are important biological control agents of numerous agricultural pests (Jalali et al. 2016).Two species of the genus *Trichogramma* were discovered and identified while surveying for egg parasitoids of the FCM *T.leucotreta* in the Israeli central coastal plain. While *T.cacaeciae* is native to Europe and widely distributed around the world (Jalali et al. 2016), the parasitoid *T.danausicida* was reported only in India (Begum and Anis 2014, Yousuf et al. 2015, Jalali et al. 2016). These two egg parasitoids are recorded for the first time in fauna in Israel. Moreover, to the best of our knowledge, this is the first report of *T.danausicida and T.cacaeciae* attacking and developing in the FCM eggs, and the first report of *T.cacaeciae* presence outside of India. The parasitism level of FCM eggs that was found in our study was very low (3.7% only in one site). Both egg parasitoids, *T.danausicida* and *T.cacaeciae*, apparently play only a minor role in keeping FCM population low in castor bean plants, and therefore are not potentially recommended biological control agents for FCM control. Similarly, Pinto et al. (2002) reported that the percentage occurrence of *T.cacaeciae* collected from parasitised tortricid eggs found on pears and apples in North America, was extremely low (less than 1%). However, our findings may contribute to better knowledge of trichogrammatids fauna in Israel and the Middle East. Since information is lacking on those two parasitoids in scientific literature, biological and ecological studies are needed to determine their biology, host list, and their impact on their host biological control.
